# Ex Vivo Hyperspectral Autofluorescence Imaging and Localization of Fluorophores in Human Eyes with Age-Related Macular Degeneration

**DOI:** 10.3390/vision2040038

**Published:** 2018-09-26

**Authors:** Taariq Mohammed, Yuehong Tong, Julia Agee, Nayanika Challa, Rainer Heintzmann, Martin Hammer, Christine A. Curcio, Thomas Ach, Zsolt Ablonczy, R. Theodore Smith

**Affiliations:** 1 Department of Ophthalmology, New York University School of Medicine, New York, NY 10016, USA; 2 Department of Ophthalmology, Icahn School of Medicine of Mount Sinai, New York, NY 10029, USA; 3 Leibniz Institute of Photonic Technology, 07745 Jena, Germany; 4 Institute of Physical Chemistry and Abbe Center of Photonics, Friedrich Schiller University Jena, 07743 Jena, Germany; 5 Department of Ophthalmology, University Hospital Jena, 07743 Jena, Germany; 6 Center for Medical Optics and Photonics, University of Jena, 07743 Jena, Germany; 7 Department of Ophthalmology, University of Alabama at Birmingham School of Medicine, Birmingham, AL 35233, USA; 8 Department of Ophthalmology, University Hospital of Würzburg, 97080 Würzburg, Germany; 9 Department of Ophthalmology, Medical University of South Carolina, Charleston, SC 29425, USA

**Keywords:** age-related macular degeneration, autofluorescence, drusen, non-negative matrix factorization, hyperspectral imaging, retinal pigment epithelium

## Abstract

To characterize fluorophore signals from drusen and retinal pigment epithelium (RPE) and their changes in age related macular degeneration (AMD), the authors describe advances in ex vivo hyperspectral autofluorescence (AF) imaging of human eye tissue. Ten RPE flatmounts from eyes with AMD and 10 from eyes without AMD underwent 40× hyperspectral AF microscopic imaging. The number of excitation wavelengths tested was initially two (436 nm and 480 nm), then increased to three (436 nm, 480 nm, and 505 nm). Emission spectra were collected at 10 nm intervals from 420 nm to 720 nm. Non-negative matrix factorization (NMF) algorithms decomposed the hyperspectral images into individual emission spectra and their spatial abundances. These include three distinguishable spectra for RPE fluorophores (S1, S2, and S3) in both AMD and non-AMD eyes, a spectrum for drusen (SDr) only in AMD eyes, and a Bruch’s membrane spectrum that was detectable in normal eyes. Simultaneous analysis of datacubes excited atthree excitation wavelengths revealed more detailed spatial localization of the RPE spectra and SDr within drusen than exciting only at two wavelengths. Within AMD and non-AMD groups, two different NMF initialization methods were tested on each group and converged to qualitatively similar spectra. In AMD, the peaks of the SDr at ~510 nm (436 nm excitation) were particularly consistent. Between AMD and non-AMD groups, corresponding spectra in common, S1, S2, and S3, also had similar peak locations and shapes, but with some differences and further characterization warranted.

## 1. Introduction

Originally deployed in satellite imagery for water resource management [1], hyperspectral imaging has found uses in food safety monitoring [2], forensic medicine [3], and biomedicine [4]. By providing spatially resolved spectral imaging, biomedical hyperspectral imaging has demonstrated utility for disease detection [5] and monitoring [6]. Hyperspectral imaging of the ocular fundus has been used in reflectance mode to study macular pigment [7] and retinal oximetry [8].

Clinical fundus autofluorescence (AF) imaging of the RPE, support cells to photoreceptors, and choroidal vasculature reveals morphologic and pathologic changes in patients with retinal diseases, including age related macular degeneration (AMD) [9,10,11]. This in vivo imaging modality acquires the total AF emission of fluorophores localizing primarily to RPE, as impacted by surrounding tissues, all excited at 488 nm wavelength. In vivo imaging can be informed by ex vivo multi excitation hyperspectral AF (HAF) imaging of human tissues [12] and analyzed by decomposition via non-negative matrix factorization (NMF) [13]. Our work so far has identified spectra and tissue localizations of individual fluorophores (or groups of fluorophores). These include three distinguishable spectra for RPE fluorophores (S1, S2, and S3) and SDr for drusen [14], the characteristic extracellular deposits of AMD, located between the RPE basal lamina and the inner collagenous layer of BrM, the inner stratum of the choroid. This suggests that a clinical HAF camera with similar capabilities, particularly for the drusen spectrum SDr could yield new diagnostic and prognostic information about AMD [15]; indeed HAF imaging is potentially one of the most precise means to analyze any retinal disease involving RPE.

Ex vivo HAF imaging for a given excitation wavelength λ_ex_ of RPE-Bruch’s membrane flat mounts (also containing drusen) records a *stack* of AF emission images, one measured at each emission wavelength λ_em_ in the range of 420 nm and 720 nm at 10 nm intervals. The resulting stack of emission data is therefore arranged by the two spatial dimensions (x, y) of the images, and the third dimension of emission wavelength λ_em_. For each point (x, y, λ_em_) in the cube, there is an associated datum, the strength of emission, in photons/sec, at that wavelength from that point in the tissue. The stack is decomposed via NMF into two matrices, a small number of spectral profiles of major fluorophores (or groups of fluorophores) and a 2D abundance image showing the tissue localization and relative strength of each major signal. The molecular identity of the fluorophores themselves may or may not be known, although the spectral profiles for some components like lipofuscin and melanolipofuscin have been described [16].

As a non-convex optimization procedure, NMF is highly sensitive to initial guesses for its values. Because the actual molecular identities of RPE fluorophores in human macula are currently uncertain, the challenge is to ensure that NMF results are physiologically meaningful. Thus, we introduced additional constraints on initializations and verified plausible tissue localization and reproducibility of the analytic solutions. This also appeared to improve the quality of the spectral profiles and interpretability of tissue localizations, represented in our pipeline as abundance images.

Ex vivo, the spectral signatures of three components of normal human RPE autofluorescence have previously been characterized: S1 and S2, believed to represent lipofuscin (LF), and S3, believed to represent melanolipofuscin (MLF) [17]. In tissues from eye donors with AMD, an additional non-LF signature that was sensitive and specific to drusen was identified as SDr [14]. Drusen are extracellular and located underneath the layer of RPE cells. In a projection fundus image or a microscopic view of flat mounted tissue, they appear under the RPE or protruding through a discontinuity in the RPE layer.

Prior multi excitation strategies used two excitations, 436 nm and 480 nm, and a user-guided algorithm (Gaussian mixture modeling) to initialize the NMF algorithm [14]. We demonstrate herein that increasing the number of excitation wavelengths from two (436 nm, 480 nm) to three, including 505 nm, seems to improve qualitative interpretability and spectral recovery. We also demonstrate an unbiased and robust automatic initialization and show NMF convergence to similar results.

Convergence of the algorithm from multiple initialization states to similar solutions speaks to robustness and reproducibility of the methodology. Quantitatively we use mean spectra and standard errors to compare the amplitudes of recovered spectral profiles graphically.

In all, we propose improvements in ex vivo HAF imaging of tissues, already shown to provide novel spectral information, to further understand and characterize AMD pathophysiology and guide future development of clinical imaging devices.

## 2. Materials and Methods

Combined RPE and Bruch’s Membranes flatmounts were prepared in two sets. The first set of seven AMD eyes was used for studying multiple excitation wavelengths and for comparing the results obtained from two different initialization methods of NMF. The two methods were user-selected Gaussians for expected spectra of RPE and Bruch’s Membrane, and novel, unbiased initialization with the first 4 non-negative singular-triplets from non-negative double singular value decomposition (NNDSVD) [18]. The second set consisted of 10 AMD eyes and 10 non-AMD eyes. AMD eyes were designated on the basis of the Alabama Age-Related Maculopathy Grading System, and images of only soft drusen were used, excluding drusen that may be the result of physiologic aging. Because the studies on the first set established the qualitative equivalence of the two initialization methods with regards to recovered spectral shapes, peaks, and abundances, we used only user-selected-Gaussians to initialize the NMF on the second set. Of note, five samples were imaged again from previous analysis using the increased number of excitation wavelengths necessary for this analysis.

Images were acquired from RPE/Bruch membrane flatmounts, prepared according to previously described methods within 4.2 h of death and preserved in 4% paraformaldehyde/0.1 M PBS [19,20], with a hyperspectral camera at 10 nm intervals from 420 nm to 720 nm (Nuance FX; Caliper LifeSciences, Waltham, MA, USA) mounted on a wide-field epifluorescence microscope (Axio Imager A2; Carl Zeiss, Jena, Germany) with band-pass filter sets for excitation at 436 nm, 480 nm, and 505 nm (Chroma Technology Corp, Bellows Falls, VT, USA), an external mercury arc light source (X-Cite 120Q; Lumen Dynamics Group, Inc., Mississauga, ON, Canada), and a 40× oil-immersion objective (numerical aperture = 1.4).

Hyperspectral AF image channels corresponding to each emission wavelength were vectored and arranged into rows of a data matrix. Multiple data matrices from corresponding excitation wavelengths were concatenated together in order of increasing excitation wavelength.

The image stacks were decomposed using NMF into spectra and spatial abundances. Because the physical tissue fluorophores have the same spatial distribution regardless of the excitation, the abundances for corresponding spectra from each excitation were also constrained to be identical for the mathematical solutions from the NMF. Thus, the *tissue abundance* for S1 will be the *same* whether the excitation used was 436 nm, 480 nm, or 505 nm, even though the *shape* of S1 itself will be different for the three excitations. We previously described results from two excitation hypercubes, [16] and we increased excitations here from two to three on the first set of eyes. Two initialization methods were also tested as described earlier.

When using NMF, non-negative constraints are vital for data sets that are innately described by non-negative numbers such as spectral emission energy or amount of a physical source. NMF allows databases of whole images to be broken down into additive parts, which are usually composed of significant, localized features. NMF is unique in that it allows for a part-based analysis as opposed to the more holistic forms of analyses achieved through methods like principal component analysis (PCA) and vector quantization (VC), which rely on linear combinations of eigenvectors and of basis images, respectively. The large proportion of vanishing coefficients generated through NMF yield sparse, basis images that can be combined [13]. And so distinct parts are pieced together to reveal the whole analogous to the way a puzzle is constructed.

At its core, NMF relies on the ability of low rank matrices to approximate large dimensions of data and consequently simplify datasets. The original non-negative matrix can be broken down into the following formula: Z=W∗H where Z is the original m × n non-negative matrix, W is the basis matrix, and H is the coefficient matrix. The rank of factorization, p, which is the number of independent basis elements in W, poses a conundrum as it must be small enough to simplify the dataset, but large enough that it can accurately approximate the dataset. In our case, this number should correspond to the number of major fluorophore spectra present in the data. Singular value decomposition (SVD) can help us find this generally agreed upon rule for choosing p: (m+n) p<mn [21]. SVD for any real or complex matrix M=UEV where U is the m × r matrix of r unit left singular vectors, E is the r × r diagonal matrix of non-negative real numbers which are the r singular values of M, and V is the conjugate transpose of the n × r matrix of r unit right singular vectors [21]. Choosing a good p< r is vital because it increases the speed of convergence and decreases the computational cost. For example, p can be selected as 3–4 and the NMF initialized with the first 3–4 non-negative singular vectors by the method of Boutidis et al. [22].

Although NMF alone cannot reveal clinically relevant information, when initialized properly with an estimated Gaussian mixture of components or SVD, the adjusted NMF in this study may reveal biologically relevant smooth peaks corresponding to the same cellular compounds or a family of compounds [23]. The recovery of spectra was verified using known fluorescence microspheres.

For the second set of images, using Gaussian initializations, we compared the amplitudes of spectra within the AMD and non-AMD groups by graphs of group means with standard errors (SEs). In this manner we also studied the congruence, that is, the similarity in peak position and shape, between spectra of fluorophores within non-AMD (Figure 5A) and AMD groups (Figure 5B), The corresponding mean spectra in common from both these groups, S1, S2, and S3, were also visually compared for qualitative assessment of differences.

## 3. Results

**Figure 1:**
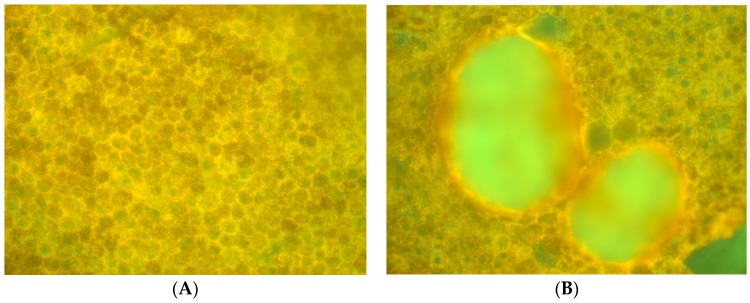
Unprocessed autofluorescence (AF) flatmount emission of normal and age related macular degeneration (AMD) eyes cited at 436 nm. (**A**) An example normal sample without AMD or any drusen. (**B**) An example AMD sample with two large drusen centrally in the image.

**Figure 2:**
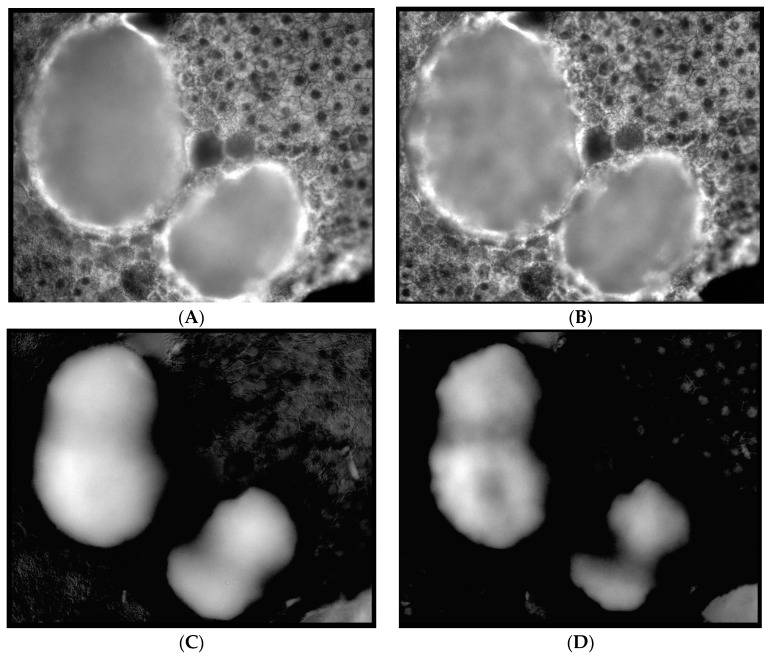
Comparison of abundance maps in an image with soft drusen achieved with two and three wavelengths of excitation. In these abundance maps, the brightness at each point is proportional to the strength of the emission of the given spectrum at that point. (**A**,**C**) Abundance maps achieved with two excitation wavelengths (436 nm and 480 nm, adapted from [14]). (**B**,**D**) Abundance maps achieved with three excitation wavelengths (436, 480 nm, and 505 nm). (**A**,**B**) Abundance maps of the retinal pigment epithelium (RPE) fluorophore spectrum S3 (**A**) and S2 (**B**). Note the high abundance of the RPE fluorophore source surrounding the dark nuclei in each cell. The distribution of fluorophore on the right may suggest attenuated RPE overlying the drusen. (**C**,**D**) Abundance maps of the drusen spectrum (SDr). The increased detail in the abundances of both RPE fluorophores and SDr obtained by going from two excitations (left) to three (right), is evident. More information is needed to discern among localizations in the *Z*-axis of this projection image.

**Figure 3:**
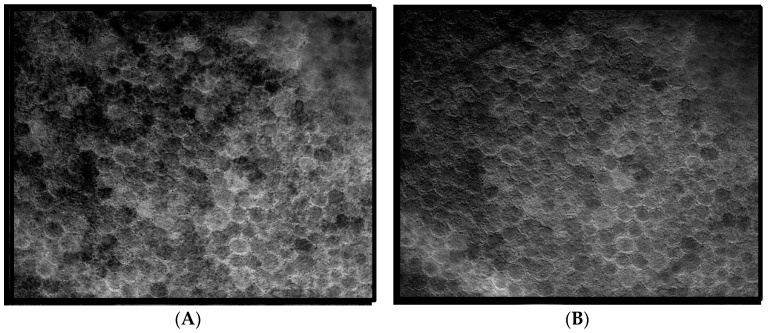

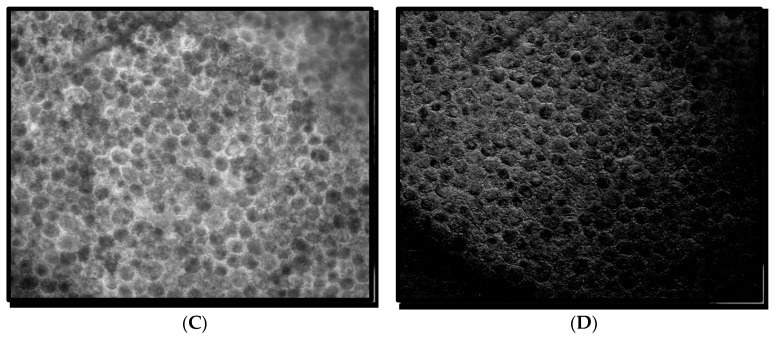
Comparison of abundance maps in a non AMD image without drusen achieved with two and three wavelengths of excitation. In these abundance maps, the brightness at each point is proportional to the strength of the emission of the given spectrum at that point. (**A**,**C**) Abundance maps achieved with two excitation wavelengths (436 nm and 480 nm, adapted from [14]). (**B**,**D**) Abundance maps achieved with three excitation wavelengths (436 nm, 480 nm, and 505 nm). (**A**,**B**) Abundance maps of the RPE fluorophore spectrum S2. Note the high abundance of the RPE fluorophore source surrounding the dark nuclei in each cell. The distribution of fluorophore on the right may suggest attenuated RPE overlying the drusen. (**C**,**D**) Abundance maps of the RPE fluorophore spectrum S3 (**C**) and Bruch’s membrane (BrM) (**D**). The similar detail in the abundances of RPE fluorophores obtained by going from two excitations (left) to three (right), is evident, and improvement is not as striking as in sample with SDr. More information is needed to discern among localizations in the *Z*-axis of this projection image.

**Figure 4:**
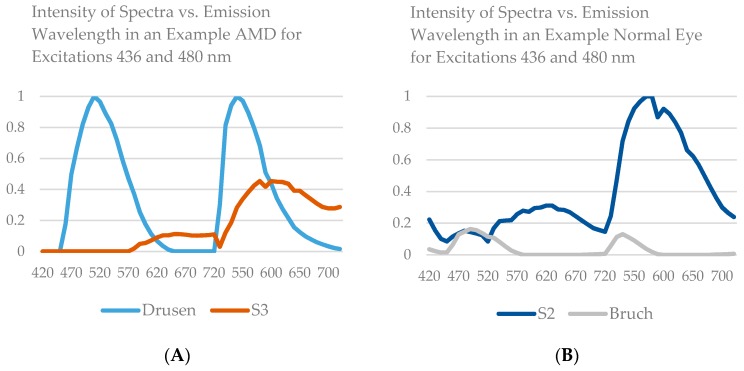

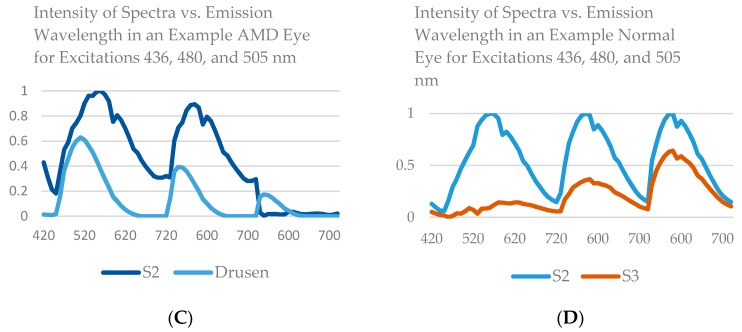
Spectral recovery of selected fluorophores from two and three wavelength excitations, and AMD and control samples corresponding to abundances in Figure 1 and Figure 2. The spectra are normalized to one. (**A**) Two wavelength excitation spectra for a normal eye corresponding to S2 (Figure 3A) and Bruch (Figure 3C). (**B**) Two wavelength excitation spectra for an AMD eye corresponding to S3 (Figure 2A) and SDr (Figure 2C). (**C**) Three wavelength excitation spectra for a normal eye corresponding to S2 (Figure 3B) and S3 (Figure 3D). (**D**) Three wavelength excitation spectra for an AMD eye corresponding to S2 (Figure 2B) and SDr (Figure 2D).

The same fluorophore families were recovered as previously described [14], that is, three spectra (S1, S2, and S3), with S1, S2 attributable to RPE LF and S3 attributable to RPE MLF and one spectrum (SDr) attributable to drusen and sub-RPE diffuse deposits. However, adding a third excitation appeared to improve the spatial and spectral recovery of the NMF algorithm. Spatially, abundance images seemed to suggest more information about fluorophore localizations within drusen deposits. In one tissue (Figure 1B) with drusen analyzed with two excitation wavelengths (Figure 2A,C and Figure 4B), the fluorophore source of S1 overlapping with drusen (i.e., sharing the same x, y but not necessarily the same z) [14] appeared to be attenuated, possibly due to out-of-plane RPE cells overlying the druse. When this tissue was analyzed with three excitation wavelengths (Figure 2B,D and Figure 4D), a more detailed, consistent pattern emerged. Spectrally, the recovered waveforms appeared less noisy, with better-defined peaks, than our previously reported with two excitation wavelengths. In a tissue without AMD and without drusen (Figure 1A), the spectral recovery and abundance images were similar with analogous peaks and spectra were recovered using both two (Figure 3A,C and Figure 4A) and three excitation wavelengths (Figure 3B,D and Figure 4C).

The change in initialization from user-selection-guided Gaussians to non-negative double singular value decomposition (NNDSVD) resulted in qualitatively similar spectra and localizations, with the advantage that NNDSVD is automatic and does not rely on user expertise and training. The convergence of the NMF algorithm to close solutions given two different initialization strategies is computationally significant, because the matrix factorization itself is underdetermined, that is, there are many possible solutions. It also gives stronger biological credibility to the results, suggesting that the recovered spectra may in fact likely represent the principal biologic fluorophores.

**Figure 5:**
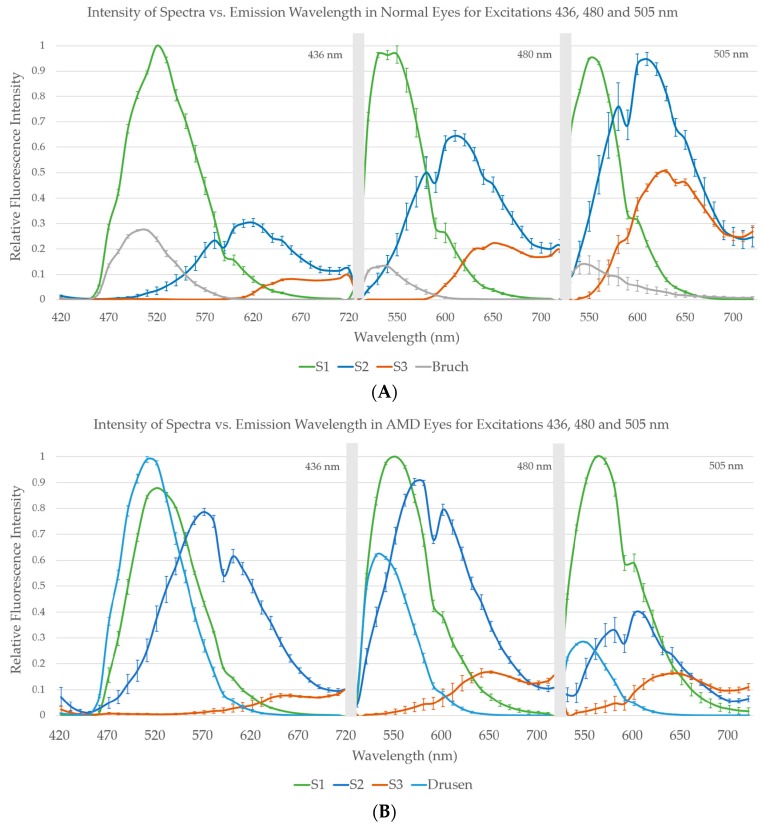
Mean spectra from 10 normal and 10 AMD eyes from three wavelength excitation AF. (**A**) Mean spectra from 10 normal eyes. Bruch’s membrane, S1, S2, and S3 mean emission spectra at excitations 436 nm, 480 nm, and 505 nm are plotted with standard error bars, normalized to the maximum emission for each excitation. Spectral amplitudes from each contributing tissue are proportional to the *total* emissions of the respective spectra from that tissue. Hence the spectral amplitudes shown here are proportional to the *mean total* emissions from all 10 tissues. (**B**) Mean spectra from 10 AMD eyes. SDr from drusen, S1, S2, and S3 mean emission spectra at excitations 436 nm, 480 nm, and 505 nm are plotted with standard error bars, normalized to the maximum emission for each excitation. BrM spectrum is less visible than in normal eyes, above.

A comparison of spectra is shown by graphs of group means with standard errors (SEs), from 10 AMD tissues and 10 non-AMD tissues. Not all spectra were recovered from each individual tissue, but within each of the groups of individual spectra—for example, all recovered S1—we found strong congruence between spectra of fluorophores within non-AMD and AMD groups (Figure 5A,B). That is, there is similarity in peak position and shape of the spectra obtained from each tissue at each excitation wavelength within a given group. The corresponding spectra in common from both these groups, S1, S2, and S3, are also similar as may be seen by visual comparison. However, there are also evident differences, as in the relative heights of the two peaks in S2. More striking, the relative strength of S3 in the non-AMD eyes is much less than S1 or S2. In addition, the small size of the data prohibits stronger conclusions. In the AMD eyes, S3 has comparable strength to S1 and S2, suggesting that MLF may be in greater abundance in AMD than in normal tissue or that the intracellular environment has changed in a way that makes this component and its underlying fluorophore(s) more visible. In AMD eyes only, the SDr spectrum was also present (Figure 5B), and the spectral peaks of SDr were particularly consistent. In non-AMD samples, another spectrum that peaked around 500 nm came from Bruch’s membrane (BrM), (Figure 5A), but was quite weak in AMD eyes (Figure 5B).

## 4. Discussion

Consistent and reproducible recovery of spectral signatures from post-mortem human retinas support the hypothesis that distinct fluorophores localize to RPE organelles, to Bruch’s membrane, and to drusen [15]. The immediate clinical advantage of this technique, if a camera were available today, is the detection of drusen and especially diffuse deposits of drusen precursors [22] that are not detectable with current AF or spectral domain optical coherence tomography (SD-OCT) imaging. Thus, HAF could provide a noninvasive method of early AMD detection and tracking in vivo and could even offer prognostic value.

We found that a spectrum localizing to BrM in normal eyes was poorly detectable in AMD eyes. This difference may be attributable to material between the RPE cell bodies and inner collagenous layer of BrM (basal laminar deposits) that attenuated this signal. Alternatively, there could be a change in the fluorophore mix or environment (e.g., extracellular pH) that caused the spectrum to fall outside the sensitivity range of the detection camera. Further analysis of cross-sectional material, in conjunction with molecular discovery techniques, should elucidate this question.

Improvements in robust spectral recovery remain the driver of progress towards development of a prognostic clinical tool. The limitations of HAF, including variability of computational solutions, continue to be minimized as techniques mature. The newer methods described in this paper should be applied to larger data sets with more robust statistical analysis as a follow up to these early findings. Our future research will include identification and development of statistical tools to assess variability in spectral peaks as well as amplitudes (Figure 3), both within individuals and between diagnostic groups. Thus, a more detailed way to describe a component spectrum could be in terms of four parameters, amplitude, peak position, width, and skewness, similar to that used in visual photopigment research, where nomograms, or standardized curves, are specified by a few parameters. Such details might be more sensitive to a shift in the microenvironment with disease, which may well result in a shift in the spectrum.

Molecular identification with imaging mass spectroscopy tied to these HAF results could be an important next step to further understand RPE physiology in health and disease and the composition of drusen for new druggable targets.
